# Stem Cells and Organoids: A Paradigm Shift in Preclinical Models Toward Personalized Medicine

**DOI:** 10.3390/ph18070992

**Published:** 2025-07-01

**Authors:** Eleanor Luce, Jean-Charles Duclos-Vallee

**Affiliations:** Unité Mixte de Recherche (UMR_S) 1193, INSERM/Université Paris-Saclay, F-94800 Villejuif, France

**Keywords:** human pluripotent stem cells, organoids, drug discovery, precision medicine, toxicology, patient-derived models, animal-free testing, pharmacology

## Abstract

**Background/Objectives**: Human pluripotent stem cells (hPSCs) and organoid technologies are transforming pharmaceutical research by providing models that more accurately reflect human physiology, genetic variability, and disease mechanisms. This review aims to assess how these systems improve the predictive power of preclinical drug development while addressing ethical concerns and supporting the advancement of precision medicine. **Methods**: We conducted a comprehensive review of the recent literature focusing on the biological principles, technological developments, and pharmaceutical applications of hPSC- and organoid-based systems. Particular attention was given to patient-derived models, integration of omics approaches, bioengineering advances, and artificial intelligence applications in drug screening workflows. **Results**: hPSC- and organoid-based platforms outperform traditional 2D cultures and animal models in replicating human-specific pathophysiology, enabling personalized drug testing and improving predictions of therapeutic efficacy and safety. These technologies also align with the ethical principles of the 3Rs (replacement, reduction, and refinement) by reducing reliance on animal experimentation. However, challenges persist, including standardization of protocols, batch-to-batch variability, and scalability. Promising solutions involve automation, high-throughput screening, and multi-omics integration, which collectively enhance reproducibility and translational relevance. **Conclusions**: Stem cell- and organoid-based systems offer a more human-relevant, ethical, and individualized approach to biomedical research. Despite current limitations, ongoing interdisciplinary innovations are expected to accelerate their clinical and industrial adoption. Collaborative efforts will be essential to standardize methodologies and fully realize the potential of these models in bridging preclinical and clinical drug development.

## 1. Introduction

The pharmaceutical industry is facing a growing need to improve the translational relevance of preclinical models used in drug discovery and development. Traditional systems such as two-dimensional (2D) cell cultures and animal models have long served as essential tools in evaluating drug efficacy and safety. However, it has been recognized for many years that, depending on the physiological processes studied, these models often fail to faithfully recapitulate human-specific responses, leading to poor predictive value and high attrition rates in clinical trials [1,2]. For this reason, there is an urgent need for more reliable, human-relevant platforms that can bridge the gap between bench and bedside.

Recent advances in stem cell biology and organoid technology offer promising alternatives to conventional 2D models, and sometimes even to the shortcomings of the animal models mentioned above. Indeed, human pluripotent stem cells (hPSCs), including embryonic stem cells (hESCs) and induced pluripotent stem cells (hiPSCs), possess the capacity to differentiate into virtually any cell type, making them powerful tools for disease modeling, drug screening, and regenerative medicine [3,4]. Moreover, the development of organoids, i.e., three-dimensional (3D) miniaturized structures that self-organize and mimic the architecture and functionality of native organs, has revolutionized in vitro modeling [5,6]. These systems not only preserve patient-specific genetic and phenotypic features, but also offer improved physiological relevance compared to 2D cultures.

The convergence of stem cell and organoid technologies has catalyzed the emergence of next-generation preclinical platforms, particularly in the context of precision medicine. Patient-derived organoids (PDOs), for example, have demonstrated utility in predicting individual responses to anticancer therapies, enabling personalized therapeutic strategies and reducing the risk of adverse outcomes [7,8,9].

However, despite the tremendous scientific advances made in this field of study over the last 10 years and their transformative potential, several challenges remain, ranging from variability in organoid generation protocols to scalability and regulatory integration [10,11]. This review aims to provide a comprehensive overview of the current landscape of stem cell- and organoid-based models in drug development, highlighting their applications, benefits, limitations, and future directions.

## 2. Human Pluripotent Stem Cells

Human pluripotent stem cells (hPSCs), including both embryonic stem cells (hESCs) and induced pluripotent stem cells (hiPSCs), possess the unique ability to self-renew quite indefinitely and to differentiate into virtually any cell type of the human body [12]. These characteristics make hPSCs highly versatile tools for studying human development, modeling diseases, evaluating drug candidates in a physiologically relevant context, and even to be used in clinical trials [13,14,15,16].

The advent of the hiPSC technology, pioneered by Takahashi and Yamanaka in 2006 [17,18], marked a paradigm shift in biomedical research by enabling the reprogramming of adult somatic cells into a pluripotent state using defined transcription factors. Compared to hESCs, hiPSCs offer notable ethical and practical advantages, particularly the non-embryonic nature of the cells and the possibility of deriving patient-specific cell lines that retain the individual’s genetic background [19,20]. This last capability is of immense value for disease modeling and precision drug testing, allowing for the study of genotype–phenotype relationships and differential drug responses in vitro [21,22].

In pharmaceutical research, hPSCs have been successfully differentiated into a wide range of relevant cell types, including cardiomyocytes [23,24], neurons [25,26], hepatocytes [27,28], and pancreatic beta cells [29,30]. These differentiated derivatives are increasingly employed in high-throughput drug screening platforms and toxicological assessments, often yielding results that more closely mirror human responses than conventional models [31]. For example, hPSC-derived cardiomyocytes have been utilized to detect cardiotoxic effects of chemotherapeutics such as doxorubicin, which may not be readily observed in non-human systems [32,33].

Furthermore, disease-specific hiPSC lines have been generated for numerous monogenic and complex disorders, including familial Alzheimer’s disease [34,35], hemophilia B [20], type 1 diabetes [36,37], and Parkinson’s disease [38,39]. These models facilitate mechanistic studies and enable screening for therapeutic compounds that target disease phenotypes at the cellular level [19,40].

While the potential of hPSCs in pharmaceutical development is considerable, certain limitations persist. Differentiation protocols often suffer from variability and incomplete maturation of derived cells [10,20]. Moreover, batch-to-batch reproducibility remains a technical hurdle, which may impact assay consistency and regulatory acceptance [41]. Nonetheless, continued optimization of differentiation strategies, combined with advances in genome-editing technologies (e.g., CRISPR/Cas9), is progressively enhancing the utility of hPSC-derived systems in drug discovery pipelines [20].

## 3. Organoids: Innovative 3D Models

Organoids represent a major advancement in in vitro modeling, providing 3D self-organizing structures that mimic the cytoarchitecture and functional characteristics of native human organs. Derived from stem cells, including adult stem cells [42], hESCs [43] or hiPSCs [28], organoids have the capacity to recapitulate complex cellular interactions, spatial organization, and organ-specific physiology in ways that traditional 2D cultures cannot [44,45,46].

The development of the organoid technology was initially driven by the work of Sato and Clevers, who demonstrated that Lgr5+ adult stem cells could give rise to long-term expanding intestinal organoids in vitro without the need for a mesenchymal niche [47]. Since then, protocols have been refined for generating organoids from a wide variety of human tissues, including the brain [48], liver [49], pancreas [50], kidney [51], lung [52], and tumor biopsies [53]. These models offer enhanced predictive power by preserving cellular heterogeneity and replicating functional compartments of organs, such as crypt–villus architecture in intestinal organoids [54] or bile canaliculi in hepatic organoids [28].

From a pharmaceutical point of view, organoids have opened new avenues for evaluating drug efficacy, toxicity, and pharmacodynamics under conditions that more closely mimic human biology. For instance, liver organoids derived from hiPSCs or adult stem cells can be used to assess hepatotoxicity, a major cause of drug attrition in clinical development [55,56], while brain organoids provide platforms for neurotoxicity testing and modeling of neurodegenerative diseases [57].

A particularly promising application of organoids deals with oncology. Patient-derived tumor organoids (PDTOs) have been shown to retain the histological and genomic features of the original tumors, including intratumoral heterogeneity and drug resistance patterns [7]. These PDTOs can be used for medium-throughput drug screening, offering real-time insight into individual responses to chemotherapy, targeted agents, or immunotherapies. Such approaches are already being piloted in clinical settings to inform treatment decisions, particularly in colorectal, pancreatic, and lung cancers [58].

Despite these advantages, several limitations must be acknowledged. Organoid cultures often lack components of the tumor microenvironment, such as immune cells, vasculature, and stromal elements, which can influence therapeutic responses. Moreover, variability in culture conditions, limited scalability, and the need for specialized technical expertise remain challenges to widespread implementation. Recent efforts to co-culture organoids with immune cells or integrate them into microfluidic “organ-on-chip” systems are helping to address some of these issues [59].

Beyond improving physiological mimicry, organoid-on-chip platforms hold significant promise for pharmaceutical applications. By combining the structural complexity of 3D organoids with the precise microenvironmental control of microfluidic devices, these systems enable more accurate modeling of human pharmacokinetics and pharmacodynamics [60,61]. In particular, hepatic organoids-on-chip are increasingly used to assess drug metabolism, hepatotoxicity, and bile canaliculi function under dynamic flow conditions that better reflect in vivo liver physiology [55]. This has direct implications for drug screening pipelines, where predicting liver-specific adverse effects remains a major bottleneck. The integration of biosensors and real-time readouts within these platforms also allows for continuous monitoring of drug responses, improving throughput and data quality [62]. As such, organoid-on-chip systems are emerging as high-value tools for pharmaceutical development, offering scalable and reproducible solutions to evaluate efficacy, toxicity, and mechanism of action with enhanced clinical relevance.

Organoid technology thus represents a transformative step forward in drug discovery and development. By offering models that closely mimic native tissue physiology and pathology, organoids serve as a bridge between traditional cell culture and in vivo experimentation, ultimately enhancing the translational relevance of preclinical testing.

## 4. Applications in Precision Medicine

The emergence of hPSCs and patient-derived organoids (PDOs) has greatly advanced the field of precision medicine, providing personalized platforms for disease modeling, drug response prediction, and therapeutic optimization (Table 1). These systems retain patient-specific genetic, epigenetic, and phenotypic features, enabling individualized approaches to treatment selection and drug development [7,63].

### 4.1. Personalized Drug Screening and Therapeutic Stratification

**Figure 1:**
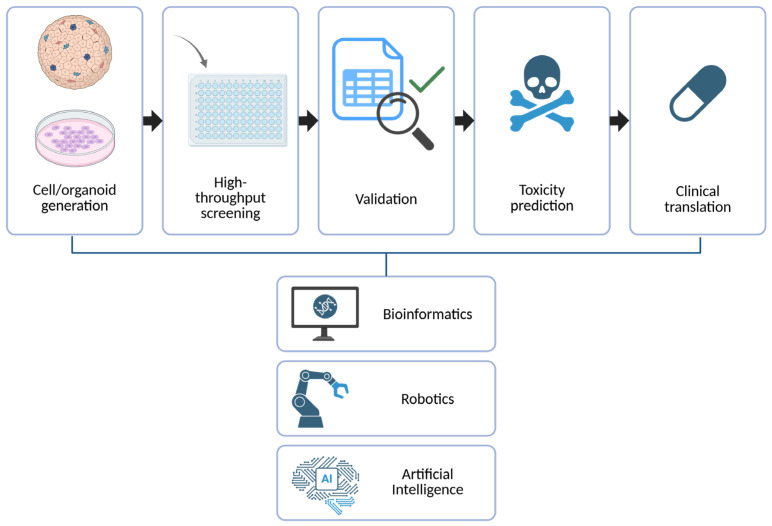
Integration of hPSCs and organoid systems into the drug discovery pipeline.

One of the most impactful uses of the organoid technology is in *ex vivo* drug screening, where PDOs derived from patient biopsies are exposed to various therapeutic agents to evaluate efficacy and resistance profiles (Figure 1). This approach has been successfully applied in multiple cancer types, including colorectal [65], pancreatic [66], breast [64], and lung cancers [67], showing concordance between organoid drug sensitivity and clinical outcomes. For example, Vlachogiannis et al. demonstrated that drug responses in PDOs from metastatic gastrointestinal cancers were predictive of patient responses, thereby highlighting their utility in guiding treatment decisions [7].

Beyond oncology, hiPSC-derived cells and organoids are being used to model rare genetic disorders and stratify patients based on expected therapeutic benefit. In cystic fibrosis, intestinal organoids derived from patient samples have been used to evaluate the efficacy of CFTR modulators in a mutation-specific manner, influencing clinical management and reimbursement policies [68].

### 4.2. Modeling Disease Heterogeneity and Genotype–Phenotype Relationships

Precision medicine depends heavily on understanding how genetic variation influences disease progression and drug response. By deriving organoids and differentiated cells from individuals with different genetic backgrounds, researchers can study interindividual variability in pharmacological profiles and disease phenotypes in vitro [69]. Models based on hiPSCs have been particularly valuable for studying monogenic diseases such as hemophilia B [20], familial hypercholesterolemia [70], and β-thalassemia [71], providing mechanistic insights and enabling personalized drug repurposing. Furthermore, advances in genome editing (e.g., CRISPR/Cas9) now allow for isogenic controls and the introduction or correction of disease-associated mutations in hPSCs. This facilitates the dissection of genotype–phenotype relationships in a controlled background and supports the development of allele-specific therapies [72].

### 4.3. Towards Clinical Integration

The integration of organoids and hiPSC-derived models into clinical decision-making is increasingly feasible due to improvements in scalability, automation, and standardization. High-throughput drug screening platforms using PDOs have already been implemented in pilot clinical trials to guide treatment selection for patients with therapy-refractory tumors [73]. In parallel, biobanks of patient-derived organoids and stem cell lines are being established to support longitudinal studies and retrospective analyses of treatment outcomes [7].

Despite their promise, challenges remain in terms of turnaround time, cost, and regulatory acceptance. Nevertheless, the growing body of clinical and preclinical data supporting the predictive accuracy of these models is fostering their adoption in personalized medicine pipelines.

## 5. Reducing and Replacing Animal Models: Ethical and Scientific Perspectives

The ethical imperative to reduce, refine, and replace animal use in biomedical research, commonly referred to as the 3Rs, has gained increasing traction in both scientific and regulatory communities. In this context, hPSC-derived models and organoids represent transformative tools that can significantly reduce reliance on animal experimentation in drug development and toxicity testing [63,74].

### 5.1. Scientific Limitations of Animal Models

While animal models have historically served as the gold standard for preclinical drug evaluation, their predictive accuracy for human outcomes remains limited. Species-specific differences in drug metabolism, immune responses, and disease progression frequently lead to discrepancies between preclinical and clinical results [75] (Table 2). For instance, only 8–10% of oncology drugs that demonstrate efficacy in animal models ultimately receive FDA approval, often due to unforeseen toxicity or lack of efficacy in humans [76,77].

Neurological, hepatic, and cardiovascular systems are particularly challenging to model accurately in animals. Stem cell-derived models offer a more human-relevant context for assessing functional responses, off-target effects, and disease-specific pathologies [56,63]. For example, hPSC-derived cardiomyocytes can reveal arrhythmogenic risks undetected in rodent models, while liver organoids provide insight into drug-induced cholestasis or steatosis under human-like metabolic conditions [56,78].

### 5.2. Regulatory Trends and Emerging Guidelines

Regulatory agencies are increasingly open to non-animal methodologies (NAMs) for safety and efficacy testing. Initiatives such as the FDA’s (Food and Drug Administration) Predictive Toxicology Roadmap and the European Medicines Agency’s support for microphysiological systems reflect a paradigm shift toward incorporating human cell-based systems into regulatory submissions (www.fda.gov; accessed on 1 June 2025). Organoids and hPSC-derived models are also central to the U.S. NIH’s Tox21 program (www.niehs.nih.gov; accessed on 1 June 2025) and the EU’s Horizon Europe initiatives (research-and-innovation.ec.europa.eu) focused on alternative testing strategies.

These trends suggest a future in which organotypic human models are validated alongside or instead of animal studies, particularly in early drug screening, mechanistic toxicology, and disease modeling. However, full regulatory acceptance will require rigorous standardization, reproducibility, and validation studies comparing these systems to historical animal data.

### 5.3. Ethical Advantages and Public Acceptance

**Figure 2:**
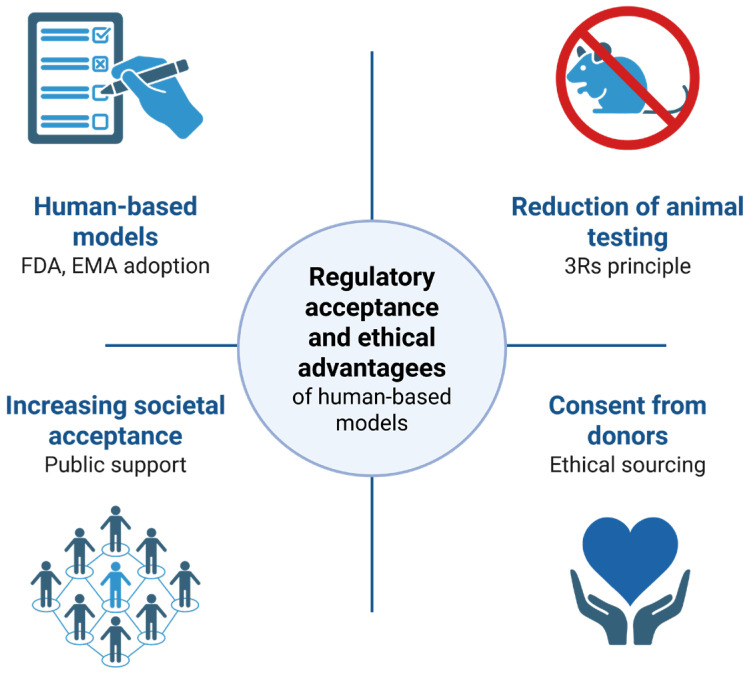
Regulatory acceptance and ethical principles.

In addition to their scientific benefits, hPSC and organoid models offer a substantial ethical advantage by minimizing the use of animals in research (Figure 2). Public support for animal-free testing is growing, especially in Europe, where citizen initiatives have prompted legislative discussions on banning animal use in cosmetics and drug toxicity studies (citizens-initiative.europa.eu). The ability to generate patient-specific models from consenting donors also aligns with ethical principles of autonomy and transparency in research.

Thus, the integration of hPSC- and organoid-based systems not only addresses scientific shortcomings of animal models, but also supports more humane and publicly acceptable research practices. These technologies are poised to become central tools in ethically aligned and human-relevant pharmaceutical research.

## 6. Current Challenges and Future Perspectives

**Figure 3:**
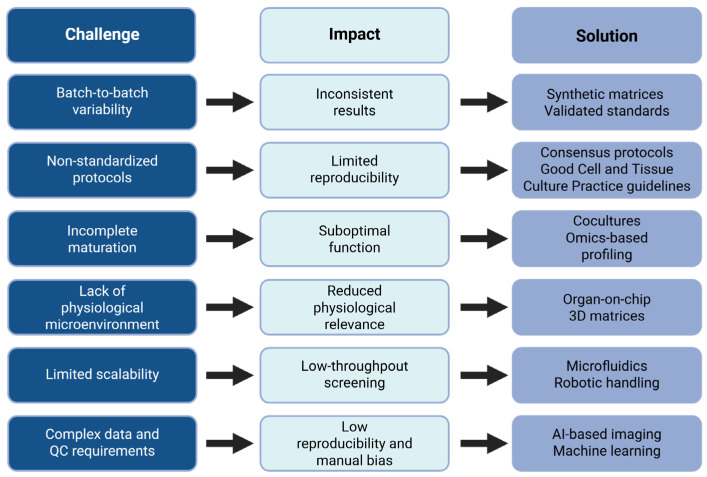
Challenges in organoid and hPSC-based platforms and emerging solutions.

Despite the rapid progress of hPSC-derived systems and organoid technologies in pharmaceutical science, several technical and translational challenges must be addressed to ensure their full integration into clinical and industrial workflows (Figure 3).

### 6.1. Standardization and Reproducibility Issues

A major limitation in the current use of hPSC-derived cells and organoids is the lack of standardized protocols for culture, differentiation, and assay development. Inter-laboratory variability in culture conditions, growth factors, extracellular matrices, and passage numbers often leads to inconsistencies in organoid morphology and function [79]. This variability can compromise reproducibility and limit the comparability of results across studies or between institutions. Moreover, batch-to-batch variability, especially in patient-derived models, remains a challenge for both preclinical testing and biobanking efforts [80]. To mitigate these issues, international consortia are now advocating for the development of consensus protocols, validated reference standards, and Good Cell Culture Practice (GCCP) guidelines [81].

### 6.2. Incomplete Maturation and Limited Functional Performance

A recurring challenge with hPSC-derived models is their incomplete maturation, which often results in suboptimal physiological functionality [20,82]. Immature cell phenotypes can limit the predictive value of drug response assays and reduce clinical translatability. To address this, strategies such as co-culture with supporting stromal or immune cells, prolonged differentiation protocols, 3D extracellular matrix-based scaffolds, and omics-guided profiling are being explored to promote more mature and functional cell states [83,84]. These approaches aim to better recapitulate in vivo tissue architecture and signaling dynamics, thereby improving the biological relevance of organoid systems.

### 6.3. Lack of Physiological Microenvironment

Organoid cultures typically lack key physiological elements such as vascularization, mechanical cues, and immune system interactions, which are essential for mimicking the native tissue context. This absence can lead to reduced physiological relevance and limit their utility in modeling systemic drug responses or disease pathogenesis. To overcome these limitations, advanced technologies such as organ-on-chip platforms, dynamic microfluidic systems, and engineered 3D matrices are being employed to recreate aspects of the in vivo microenvironment, including perfusion, mechanical stress, and multicellular interactions [85,86]. For instance, vascularized organoid-on-chip platforms integrating endothelial networks have been shown to enhance long-term organoid viability, maturation, and functionality [87]. Similarly, microfluidic devices incorporating perfusable vasculature significantly improve nutrient–oxygen delivery and structural integrity in brain organoid models [88]. Moreover, biomechanically informed designs that include shear stress and ECM elasticity cues further promote physiologically relevant vascular network formation within organoids [89].

### 6.4. Integration of Omics Technologies and Artificial Intelligence

The complexity of stem cell-derived and organoid-based systems demands advanced tools for characterization and quality control. Multi-omics approaches, including single-cell transcriptomics, proteomics, metabolomics, and epigenomic profiling, enable comprehensive phenotyping and mechanistic insights into drug responses [90]. These datasets can uncover subtle biological differences between organoids derived from different individuals or disease states, enhancing precision modeling. In addition to maturation assessment, omics-based profiling is increasingly used to monitor differentiation fidelity and identify key regulatory pathways driving tissue functionality.

Simultaneously, artificial intelligence (AI) and machine learning (ML) tools are being used to manage and analyze the high-dimensional data generated from organoid assays [91]. AI-based image analysis improves the quantification of organoid growth, morphology, and drug response, while ML models can predict differentiation trajectories and classify phenotypes based on transcriptomic signatures [92]. These technologies have the potential to automate and scale organoid-based screening platforms, thus improving throughput and objectivity [93].

### 6.5. Toward High-Throughput, Automated Screening Platforms

Scalability remains a significant bottleneck in translating stem cell- and organoid-based assays into high-throughput drug discovery pipelines. Traditional culture systems are labor-intensive and low-throughput, limiting their utility for pharmaceutical applications. Recent advances in microfluidics, bioprinting, and organ-on-chip platforms are enabling miniaturized, multiplexed, and automated systems for drug testing [94]. For instance, droplet-based microfluidic systems allow parallel culture and treatment of hundreds of organoids with minimal reagent use [95]. Furthermore, robotic liquid handling and AI-driven imaging platforms are being developed to perform automated compound screening on organoid arrays, reducing operator bias and increasing efficiency [96].

These integrated technologies, when combined with cloud-based data infrastructure and standardized data formats, could lead to next-generation screening systems that are both human-relevant and compatible with regulatory expectations.

## 7. Conclusions

Human pluripotent stem cells (hPSCs) and organoid technologies have emerged as transformative tools in pharmaceutical research, enabling more accurate, personalized, and ethical models of human physiology and pathology. These platforms offer substantial improvements over traditional models by faithfully reproducing human-specific cellular functions, genetic backgrounds, and organ-level architecture. As such, they are increasingly integrated into drug discovery pipelines, disease modeling efforts, and precision medicine frameworks.

Organoids and hPSC-derived systems have already demonstrated their value in predicting drug efficacy and toxicity, modeling complex diseases, and tailoring treatments to individual patients. In parallel, they offer a promising alternative to animal testing, addressing long-standing concerns about translational failure, species differences, and ethical acceptability. Advances in CRISPR/Cas9 genome editing, microfluidics, and co-culture technologies continue to expand their capabilities, allowing for more sophisticated models that include immune, stromal, and vascular components.

Despite these advancements, several challenges must be overcome to ensure broader adoption and regulatory acceptance. These include improving reproducibility and standardization, scaling up production for high-throughput applications, and validating the predictive performance of these models across diverse therapeutic areas. Close collaboration between academic researchers, industry stakeholders, and regulatory agencies will be critical to developing consensus protocols and quality benchmarks.

Looking ahead, the integration of organoids and hPSC-based models with artificial intelligence, patient-derived biobanks, and organ-on-chip systems is likely to further accelerate the transition toward a more predictive, personalized, and humane drug development paradigm. These technologies represent not just a refinement of the existing models, but a foundational shift toward truly human-centered biomedical research.

## Figures and Tables

**Table 1:** Summary of applications of hPSC and organoid-based models in pharmaceutical research.

Application Area	Model Type	Advantages	Limitations
Drug efficacy screening	Organoids hPSC-derived cells [7,31]	Human-specific responses Patient-tailored	Cost Technical complexity
Toxicity testing	hPSC-derived hepatocytes/cardiomyocytes [56]	Better prediction of human toxicity	Limited maturity of differentiated cells
Disease modeling	iPSC-derived models Organoids [20]	Genetic accuracy Chronic disease modeling	Time-intensive derivation
Personalized therapy selection	Patient-derived organoids (PDOs) [64]	Reflects patient heterogeneity Fast screening	Limited scalability Requires biopsy
Animal replacement	All hPSC/organoid models [5]	Ethical Human-relevant Scalable	Regulatory acceptance still developing

**Table 2:** Comparison of preclinical models for drug development.

Criteria	2D Culture	Animal Model	Organoid
Architecture	Flat	Organism level	3D tissue “organ-like” level
Human relevance	Low	Medium	High
Cost	Low	High	Medium
Clinical predictivity	Low	Medium	High
Reproducibility	High	Medium	Medium

The values “low”, “medium”, and “high” are based on comparative assessments from published reviews and expert consensus in the field. Rankings are indicative rather than strictly quantitative and aim to help readers contextualize the strengths and limitations of each model.

## Data Availability

No new data were created or analyzed in this study. Data sharing is not applicable to this article.

## Abbreviations

The following abbreviations are used in this manuscript:

PSC	Pluripotent stem cell
PDO	Patient-derived organoid
PDTO	Patient-derived tumor organoid
ESC	Embryonic stem cell
iPSC	Induced pluripotent stem cell
